# Shape Memory Polyurethane with Porous Architectures for Potential Applications in Intracranial Aneurysm Treatment

**DOI:** 10.3390/polym11040631

**Published:** 2019-04-05

**Authors:** Jingyu Wang, Robert Kunkel, Jishan Luo, Yuhua Li, Hong Liu, Bradley N. Bohnstedt, Yingtao Liu, Chung-Hao Lee

**Affiliations:** 1 School of Aerospace and Mechanical Engineering, The University of Oklahoma, Norman, OK 73019, USA; jingyuwang@ou.edu (J.W.); rob.kunkel-1@ou.edu (R.K.); jishanluo@ou.edu (J.L.); 2 Institute of Biomedical Engineering, Science and Technology (IBEST), The University of Oklahoma, Norman, OK 73019, USA; liu@ou.edu; 3 School of Electrical and Computer Engineering, The University of Oklahoma, Norman, OK 73019, USA; yhli1500@ou.edu; 4 Department of Neurosurgery, The University of Oklahoma Health Sciences Center, Oklahoma City, OK 73104, USA; bradley-bohnstedt@ouhsc.edu

**Keywords:** polyurethane, shape memory, porous architecture, glass transition temperature, thermo-mechanical properties, electrical resistance heating, shape recovery activation, micro-CT

## Abstract

Conventional endovascular embolization of intracranial (or brain) aneurysms using helical detachable platinum coils can be time-consuming and occasionally requires retreatment due to incomplete coil packing. These shortcomings create a need for new biomedical devices and methods of achieving brain aneurysm occlusion. This paper presents a biocompatible and highly porous shape memory polymer (SMP) material with potential applications in the development of novel endovascular devices for treating complex intracranial aneurysms. The novel highly porous polyurethane SMP is synthesized as an open cell foam material with a glass transition temperature (*T*_g_) of 39 °C using a sugar particle leaching method. Once heated above the *T*_g_, the compressed SMP foam is able to quickly return to its original shape. An electrical resistance heating method is also employed to demonstrate a potential triggering design for the shape recovery process in future medical applications. The mechanical properties of the developed SMP foam are characterized at temperatures up to 10 °C above the respective *T*_g_. The results from this work demonstrate that the porous SMP material developed in this study and the electrical resistance heating trigger mechanism provide a solid foundation for future design of biomedical devices to enhance the long-term therapeutic outcomes of endovascular intracranial aneurysm treatments.

## 1. Introduction

Polymeric materials with suitable thermo-mechanical properties and functionality, such as biocompatibility, biodegradability, oxidative resistance and shape memory/recovery, are clinically desirable for biomedical applications. In the past decade, the advancement of polymer science has led to the development of shape memory polymers (SMPs) for use in the design of novel biomedical actuators, sensors, devices and smart systems [1,2,3,4], among others. SMPs can maintain a temporary shape when they are cooled below their glass transition temperature. Once appropriate thermal, electrical, or environmental conditions are satisfied, SMPs autonomously recover from this temporary shape to their original geometry without any external forces. This phenomenon is commonly referred to as shape recovery. It has been shown that the shape recovery and relaxation features of SMPs are associated with the intrinsic elastic deformation stored in their polymeric networks during prior manipulation [5,6,7]. In general, SMPs possess many desirable qualities for medical use: The ability to undergo large elastic deformation, low weight, low cost, an easily controllable synthesis procedure, and the potential for biocompatibility.

Several shape recovery activation methods for SMPs, including thermal energy [8,9,10], light [11,12], and moisture [13,14], have been reported in literature. Thermally-induced shape recovery is the most popular method owing to the simplicity of generating, applying, and controlling environmental temperature using laboratory equipment or other available heat tools under clinical settings. Vernon and Vernon published the first thermally-induced SMP in 1941, demonstrating that a dental material made of methacrylic acid ester resin possessed good elastic memory and was able to recover to its original shape after enough heat was applied [15]. By now, thermally-induced SMPs include polyurethanes [8,16], polyetheresters [17,18], polynorbornene [1,19], and poly(styrene-block-butadiene) [20,21]. Nanoparticles, such as carbon nanotubes and graphene oxide, have been dispersed within SMPs to fabricate shape memory nanocomposites for biomedical applications [22,23,24]. More detailed information about emerging SMP formulations and synthesis methods can be found in recent review papers on SMPs [25,26,27,28].

Novel medical devices that take advantage of the unique properties of SMPs have been developed in the last decade that include clot removal devices, intracranial aneurysm (ICA) occlusion devices, and vascular stents [1,2,29]. Most reported SMP-based medical devices were thermally activated, either directly through a heating element, or through photo-thermal and electrical-thermal approaches. Aneurysm occlusion devices have been one of the most popular applications for biocompatible SMPs. As the current gold standard of intracranial aneurysm treatment, endovascular embolization using Guglielmi detachable coils (GDCs) aims at complete and lasting occlusion [29,30,31]. However, long-term treatment outcomes of the GDC-based neurosurgery have been shown to be less satisfactory than expected: The recurrence rate of completely embolized aneurysms could approach 41% within 3–5 years of initial therapy, with 26% of those requiring retreatment, increasing the health care burden [32,33]. SMP materials could present an alternative approach to GDC-based endovascular coil embolization, since their unique shape recovery property allows for a similar style of deployment and occlusion. SMP foams in particular are of interest because of their easily controllable porosity, which allows for consistent volume fill ratios—a property that varies for coil embolization [34].

Since a wide variety of materials fall under the SMP category, the current materials under investigation for SMP embolization treatments are quite diverse. Materials, including polypropylene glycol, polyether polyol polyurethane, epoxy, and aliphatic polyurethane, have been used to synthesize SMP foams with varying mechanical properties [35,36,37,38,39,40]. An open-cell porous polyurethane SMP material referred to as Cold-Hibernated Elastic Memory (CHEM) foam has been investigated for the fabrication of aneurysm occlusion devices. Metcalfe et al. reported a series of experimental investigations and an animal study using SMP foams for aneurysm treatment [41]. Their results showed that the open cellular structure of the SMP foams increased the growth of cells in neointima formation. Further biocompatibility studies performed by Farè et al. showed that the material has good compatibility with human fibroblasts despite having a low affinity for platelet adhesion [42]. In addition to aneurysm treatment, Sokolowski et al. suggested that this material could be used for thrombus removal, arterial grafts, braces, splints, prosthetics, implants, and both soft and hard tissue engineering scaffolds [5]. Recently, low-density aliphatic polyurethane SMP foams manufactured with a chemical blowing process have been used to create uniformly-shaped embolization plugs, which demonstrated stable thrombus formation and complete endothelialization of the aneurysm neck within 90 days [43]. The same polyurethane SMPs have been applied as a coating on platinum coils in a modified version of coil embolization, delivering promising results through in vitro experiments and in vivo animal studies [43,44,45]. However, even with SMP-enhanced coils, the risk of coil compaction and aneurysm recanalization still present clinical and technical challenges due to resorption of the polymer coating.

In this paper, we present the synthesis procedures and characterization of an SMP foam created using a sugar particle leaching technique. The synthesis method used in this study enables fine control over both the pore size and the density of the synthesized porous foam—a characteristic enabling tunable mechanical properties such as stiffness and compression ratio for device design applications. The porous microstructure of the SMP foam was characterized using both scanning electron microscopy (SEM) and micro computed tomography (micro-CT). We also report our experimental procedures for the characterization of the foam for a certain range of pore sizes and a single monomer composition. Although the testing in this study was conducted on a single group of identical samples, both the pore size and monomer composition can be judiciously adjusted during synthesis to achieve desired mechanical and thermomechanical properties respectively [46]. The mechanical performance of the porous SMP was evaluated up to 10 °C above the SMP’s glass transition temperature using uniaxial compressive loading. Finally, we demonstrate a potential deployment method using a group of resistively heated carbon fiber filaments within compressed SMP foam to raise its temperature and activate shape recovery.

## 2. Materials and Methods

### 2.1. Materials

In this study, the following three monomers were used to synthesize aliphatic polyurethane shape memory polymers: (i) Hexamethylene diisocyanate (HDI, ≥99.0%), (ii) *N*,*N*,*N*′,*N*′-tetrakis (hydroxypropyl) ethylenediamine (HPED, ≥98.0%), (iii) Triethanolamine (TEA, ≥99.0%). The monomer content percentages by weight of HDI, HPED, and TEA used for all synthesis reactions were 60.7%, 10.6%, and 28.7%, respectively [46,47]. All the materials were purchased from Sigma Aldrich (St. Louis, MO, USA).

### 2.2. Preparation of Solid and Porous SMP Materials

To synthesize solid, or pristine, SMPs, HDI, HPED, and TEA were first measured in the weight ratio described above and mixed using a high-speed shear mixer for 5–6 min, and then cast into their ASTM D638 Type V dog-bone or 45 mm by 8 mm by 1 mm rectangular molds for curing. The materials were degassed three times and protected under a nitrogen environment before and during curing using a vacuum oven (Being BOV-20, Being Instruments Inc., Riverside, CA, USA). The temperature profile was first kept at room temperature for 1 h, followed by a ramp of 9.6 °C per hour up to 130 °C, then held for 1 h at 130 °C, and finally cooled back to room temperature. The fully cured SMP samples were removed from the molds, sealed in vacuum bags, and stored in a vacuum desiccator (Bel-Art Lab, SP Scienceware, Wayne, NJ, USA) to ensure no moisture contamination occurred before subsequent thermomechanical characterization experiments [48].

**Figure 1:**
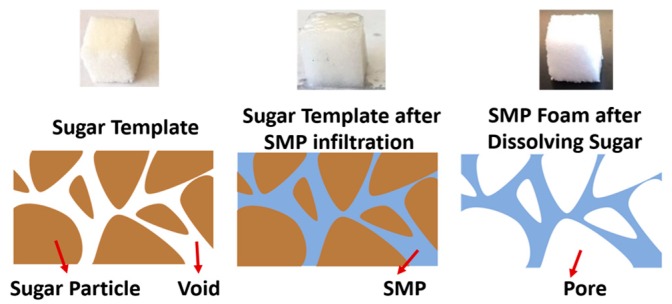
Schematic of the sugar particle leaching method for fabrication of the porous shape memory polymer (SMP) foam.

Novel highly-porous SMP foams were synthesized using a sugar particle leaching method, as depicted in Figure 1. In brief, sugar templates in the shape of cubes and thin sheets were first manufactured by compressing an appropriate amount of pure cane sugar (Florida Crystals Inc., West Palm Beam, FL, USA), purchased from a local food store, into a silicon rubber mold. The average sugar particle size was measured using scanning electron microscopy and was found to be about 500 μm. A slight amount of water was added with granulated cane sugar to improve the formability and sugar’s interfacial bonding. The water was lightly misted onto the surface of a spatula using a spray bottle, and then stirred into the mixture. This step was repeated until the sugar grains were observed to stick to one another when scooped with the spatula. The sugar was then compressed into a mold and dried in a vacuum oven at 130 °C for 2 h.

Once the sugar templates were prepared, the 3 monomers with the above-mentioned content percentages were measured and mixed by a high-speed shear mixer for 5–6 min. The sugar templates were then immersed in the mixed monomer solution and were kept in vacuum in a freezer at –5 °C for 24 h. The monomers completely infiltrated through the sugar template, partially reacted, and were then post-cured following the heating procedure as previously described for curing the solid SMP specimens. The fully-cured SMP/sugar templates were next immersed in de-ionized (DI) water and kept in a bath sonicating for 1 h to fully dissolve all the cane sugar. The manufactured porous SMP samples were kept in a vacuum oven at 50 °C for 24 h to fully eliminate all the humidity trapped in the foam, and then stored in a vacuum desiccator (Bel-Art Lab, SP Scienceware, Wayne, NJ, USA) before subsequent experiments.

### 2.3. Experimental Characterizations—Investigations of Microstrcutural Morphology, Shape Recovery, and Mechanical Performance

The *T*_g_ of the synthesized SMP has been characterized using standard dynamic mechanical analysis (DMA; TA Q800, TA Instruments, New Castle, DE, USA) and differential scanning calorimetry (DSC; TA Q50, TA Instruments, New Castle, DE, USA) tests. The detailed DMA and DSC characterization procedures and results have been reported in our previous study [46]. The shape recovery capability of the synthesized porous SMP material was qualitatively and quantitatively investigated using a direct heating method. The SMP foam sample was first heated on a hot plate at 70 ℃ for 2 min, compressed, and cooled back to room temperature in air. Then, the sample was placed on a hot plate with a surface temperature of 70 °C again to investigate its shape recovery process.

The microstructure, pore size, and the porosity of the porous SMP foam were characterized by a TESCAN SEM system (TESCAN Co., Brno-Kohoutovice, Czech Republic) and a micro-CT imaging system (c.f. Appendix A). The synthesized SMP foam was first sputter coated to improve the surface conductivity and prevent charging before taking the SEM images. SEM images were taken from the top surface and the cross-section in the middle of the cubic SMP foam samples. To include enough pores in each SEM image, relatively low magnifications of 50–100 times were used for all the acquired SEM images. The SEM images were then analyzed using ImageJ software (National Institute of Health, Bethesda, MA, USA) to obtain the average pore size of the SMP foam.

Following previous studies in literature [49,50], the porosity of the SMP foam was roughly estimated using Equation (1).

(1) $$Porosity=1-\frac{\rho_{\text{porous SMP}}}{\rho_{\text{solid SMP}}}$$

where $\rho_{\text{solid SMP}}=M_{\text{solid cube}}/V_{\mathrm{cube}}$ and $\rho_{\text{porous SMP}}=M_{\text{porous cube}}/V_{\mathrm{cube}}$ are the “apparent densities” of the solid SMP cube (10 mm by 10 mm by 10 mm) and the porous SMP cube, respectively. The masses of both the solid and porous SMP specimens ($M_{\text{solid cube}}$ and $M_{\text{porous cube}}$, respectively) were measured by a digital scale and the volume was determined based on the dimensions of the cube. Ten samples were measured for both solid and porous SMP to guarantee accuracy and repeatability.

**Figure 2:**
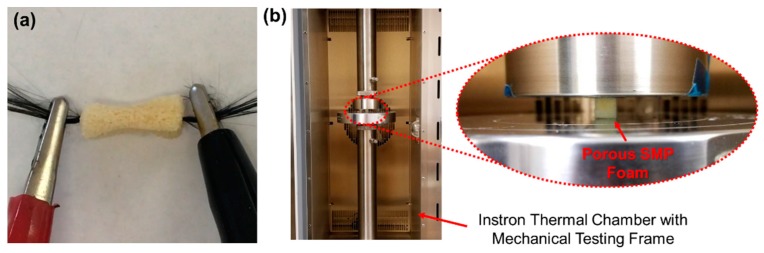
(**a**) Experimental photo of the electrically-heated procedure to activate the shape recovery of the SMP foam using carbon fiber wires. (**b**) Image of the experimental setup in thermal chamber for mechanical characterization of the SMP foam.

Electrically heated carbon fiber filaments were used to simulate a potential deployment configuration for the SMP (Figure 2a). An arbitrarily sized 10 mm by 10 mm by 15 mm SMP sample was synthesized according to the procedures described above, and a bundle of carbon fibers was introduced through the center of the sample using a needle, resulting in a foam sample with a simple integrated heating element. The foam was heated above its *T*_g_, compressed manually, and then cooled at room temperature to fix the temporary compressed shape. Direct current (DC) at 0.5, 1.0, 1.5, and 2.0 amperes was applied on the carbon fiber wires to generate the required heat and trigger the shape recovery of the compressed SMP foam, by using an electrical resistance heating method. The electrical heating was current controlled, and due to the high conductivity of the carbon fibers used in this test, applied voltages were 0.2 and 0.8 Volts. The surface temperature was measured using an infrared (IR) camera every 10 s, and thermal images were captured at each time point for illustration. The electrical resistance heating speed and magnitude were quantified by controlling the electrical current applied to the porous SMP samples.

The mechanical behavior of the porous SMP samples was characterized using an Instron 5969 dual-column mechanical testing system (Instron Co., Norwood, MA, USA) integrated with an environmental chamber (Figure 2b). Cubic SMP foam samples (10 mm by 10 mm by 10 mm) were tested until failure at room temperature (*n* = 3). Then, the failure strain was used to conduct cyclic compressive testing up to 40% of the failure strain (~90%) at a rate of 2 mm/min. Key mechanical properties were measured under 10 compressive load cycles at room temperature (25 °C, *n* = 3), *T*_g_ of SMP foam (39 °C, *n* = 3), and *T*_g_ + 10 °C (49 °C, *n* = 3). All the samples were pre-heated in the environmental chamber at the required temperature for 30 min before any mechanical tests.

## 3. Results and Discussions

### 3.1. Shape Memory and Recovery Features of the Synthesized SMPs

DMA tests showed the *T*_g_ of synthesized SMP was 37.2 °C, and DSC tests showed the *T*_g_ was 39 °C. It is typical to see a slight difference between DSC and DMA tests due to the different testing mechanisms. Since the developed SMP is being designed to treat aneurysms as a biomedical device, the obtained *T*_g_ slightly above normal body temperature is optimal and appropriate when designing a device that can deploy without causing tissue damage [51,52].

**Figure 3:**
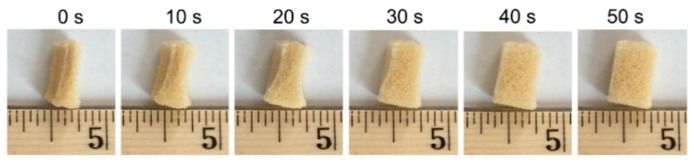
Progression of the shape recovery process of the compressed porous SMP foam in response to direct heating above the SMP’s *T*_g_.

As shown in Figure 3, the SMP foam started recovering from the compressed shape after 10 s. In 50 s, the shape of the SMP foam was completely recovered to its normal cubic shape. The fast shape recovery (<1 min) demonstrates that the synthesized SMP foam has the potential to quickly recover from a large deformation, making this SMP material a desirable candidate for the design of catheter-deployed embolic devices. When the SMP is placed on a hot plate set to the range of 38–45 °C required for endovascular embolization procedure, we observed that the compressed SMP foam can fully recover to its original shape within two minutes. This simplified, idealized test was performed in an open-air lab environment without controls for humidity.

A significant difference in color was observed between the pristine solid SMP and the porous SMP specimens. While the pristine solid SMP was primarily transparent (c.f. Appendix B and Figure A2), turning white with sanding after synthesis, the SMP foam turned a beige-yellow color during preparation. Yellowing of polyurethane foam is a common occurrence that can result from any combination of three typical causes. The first type of yellowing occurs because of the formation of quinones (yellow in color) after exposure to UV light. This reaction primarily occurs in polyurethanes synthesized with aromatic isocyanates, and ours is aliphatic isocyanates, so we do not suspect that this reaction is present. The second type of yellowing occurs when the foam is oxidized by gasses (usually nitrogen oxides or ozone), and the third type occurs when the foam is exposed to high heat [53].

During synthesis, we noticed a decrease in yellowing when we performed the reactions in a pure nitrogen environment rather than in air, as well as when we lowered the temperature level to dissolve sugar and dry the foam. The yellowing that still occurs is likely due to the applied heat during our sugar dissolution and drying procedures, since we do not believe there is a significant source of oxidative gas during any of our synthesis procedures. There is also the possibility of an unforeseen interaction between the SMP monomer and sugar that occurs before curing, potentially contributing to the change of the surface color.

### 3.2. Microstructural Analysis

**Figure 4:**
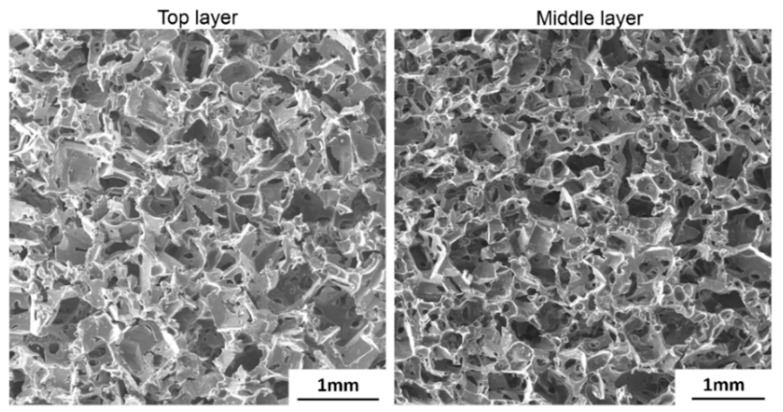
SEM images taken from the top layer and the middle layer of the porous SMP foam fabricated using the sugar particle leaching method, showing the open-cell structure of the foam.

Comprehensive microstructural quantifications of the synthesized SMP foam were carried out to understand the foam’s properties. The fabricated SMP foam was examined using SEM to measure the average size of open pores, which was a critical parameter to evaluate the density and compression capability of the synthesized SMPs foam. More than 10 SEM images were taken from either the top surface of the SMP foam or the cross-section cut in the middle of the SMP foam. Typical SEM images were shown in Figure 4. The average pore size is about 480 μm. No sugar particles were observed in any of the acquired SEM images, indicating the complete removal of cane sugar during the sonication process.

**Figure A1:**
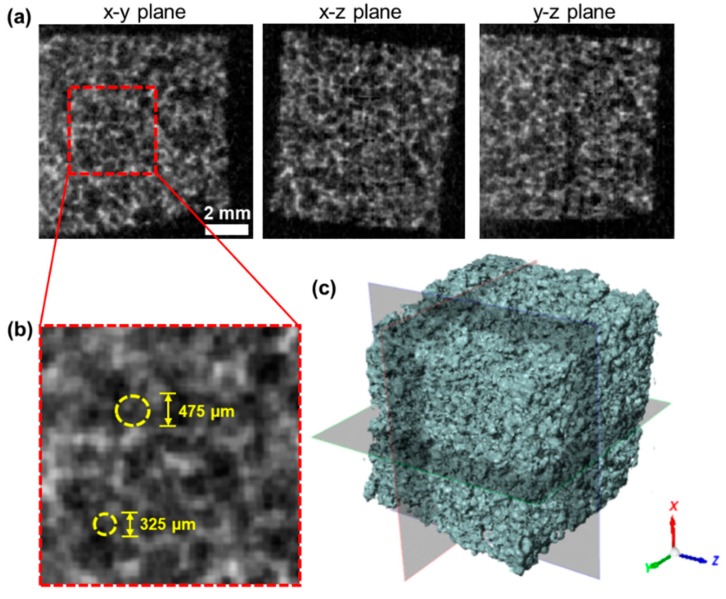
Micro-CT images showing the microstructure of SMP foam: (**a**) 2D image slices on orthogonal planes, (**b**) zoom-in area of the 2D image slice on the *x-y* plane, demonstrating the typical pore sizes of the SMP foam, and (**c**) the visualization of the reconstructed 3D geometry of the cubic SMP foam (10 mm by 10 mm by 10 mm).

**Figure A2:**
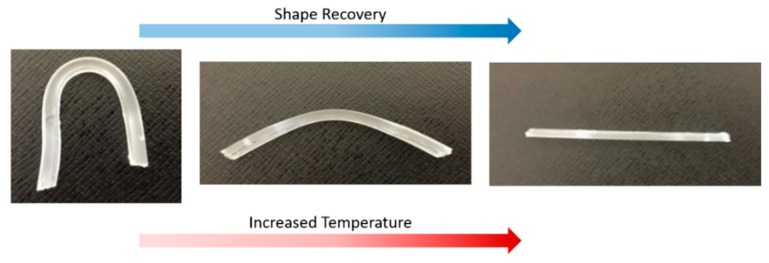
Shape recovery of the bent solid SMP beam (temporary shape) into the originally programmed straight shape after heating above the SMP’s *T*_g_.

On the other hand, representative 2D and 3D micro-CT images of entire porous SMP foam are shown in Figure A1a,b. The 2D micro-CT scans were carried out on the x-y, x-z, and y-z planes individually. The typical pore size is shown in the 2D micro-CT images (Figure A1b), and generally agrees with the measurements obtained through the SEM imaging. The slices of 2D micro-CT images were compiled to create the 3D micro-CT image, as shown in Figure A1c. The 3D images can be used for future computational modeling and optimization of the porous SMP forms.

The average density of the solid SMP was 1.172 g/cm^3^, and the average density of the porous SMP foam was 0.168 g/cm^3^. Therefore, the average porosity of the 10 measured SMP foam samples (*n* = 10) was 85.7%. It is expected that this high porosity will allow significant elastic deformation of SMP at temperature above *T*_g_, which can allow for the delivery of embolic devices for the treatment of a wider range of intracranial aneurysm sizes.

### 3.3. Electrical Resistance Heating-Based SMP Triggering Method

**Figure 5:**
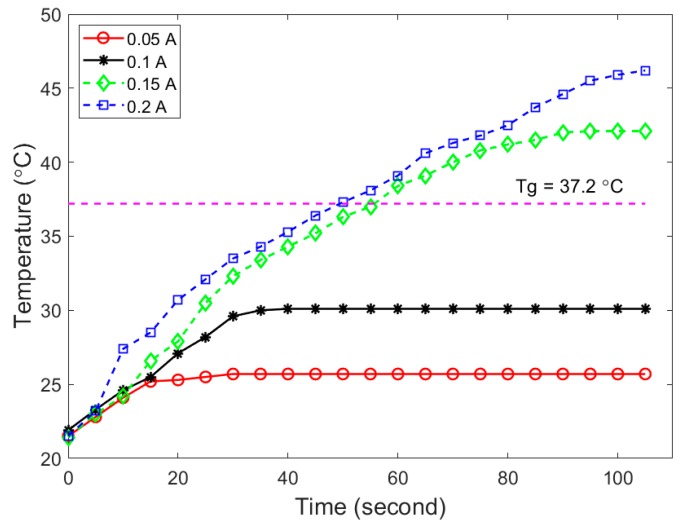
Surface temperature of the SMP foam specimen at various applied electric current magnitudes during the electrical resistance heating.

**Figure 6:**
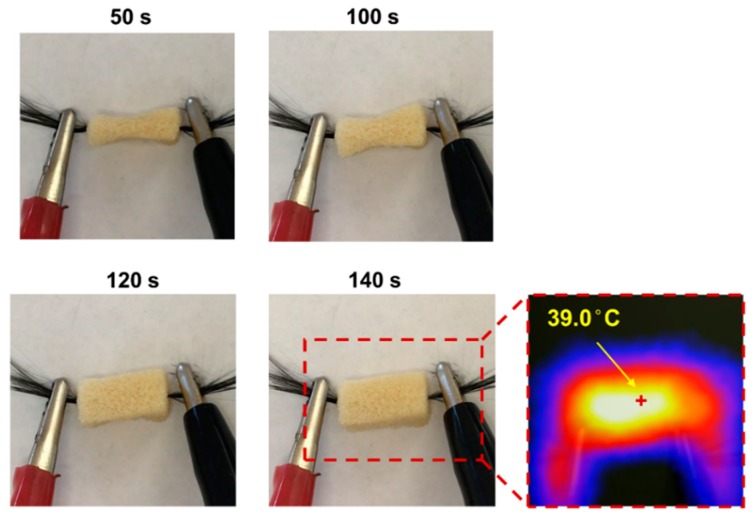
Experimental photos of the shape recovery process of the compressed SMP foam under 0.15 A DC, along with the measured surface temperature profile via an IR camera at 140 s after the applied electric current.

An appropriate triggering method is necessary to successfully deploy the highly compressed SMP foam for the aneurysm occlusion application. Electrical resistance heating was used in this study owing to its ease of implementation for potential surgical applications. Although minimal DC is preferred to improve the patient’s safety in the surgical environment, it is noted that the SMP sample was not able to reach the required *T*_g_ when 0.05 A and 0.1 A DC were applied during the electrical resistance heating process (Figure 5). The equilibrium temperature levels measured on the SMP surface were 25.7 °C and 31.3 °C when 0.05 A and 0.1 A DC were applied, respectively. Once the applied DC current increased to 0.15 A, the temperature of the entire SMP foam was able to increase above its *T*_g_ within 120 s. The measured equilibrium surface temperature on SMP sample was 43.2 °C and 47.8 °C when 0.15 and 0.2 A DC were applied, respectively. Since human vessels can be permanently damaged from short exposure (<5 min) to temperatures above 45 °C [51,52], the optimal DC is selected to be 0.15 A for this particular test. More detailed fine tuning of the applied DC can be carried out at the stage of future in vitro study and refined again before in vivo animal studies, so that the required *T*_g_ of the SMP can be quickly achieved and the deployed temperature is still kept in the safe range for surgical applications. It is also worth noted that although the heating element operates with very low voltages, the currents considered in this demonstration have the potential to seriously harm the patient in a treatment scenario without the proper safety considerations in place. The demonstrated deployment technique is merely intended to prove the possibility of SMP deployment around a heating element, rather than proposing a clinical application-ready prototype. The progression of the SMP’s shape recovery during a 0.15 A DC deployment trial, along with a visualization of the sample’s surface temperature measured using an IR camera, is illustrated in Figure 6.

### 3.4. Mechanical Characterization of the SMP Foam Under Compressive Loading

**Figure 7:**
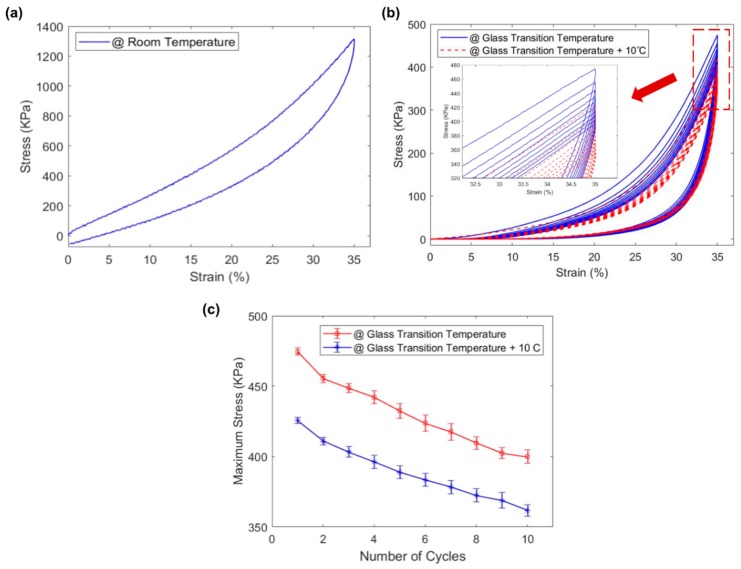
Mechanical characterizations of the fabricated SMP foam under the cyclic compressive loading conditions: (**a**) the typical mechanical response of the SMP foam at room temperature; (**b**) representative mechanical responses of the SMP foam at *T*_g_ and *T*_g_ + 10 °C under cyclic compressive loading, and (**c**) comparison of the maximum stress at the 35% strain for various loading cycles at *T*_g_ and *T*_g_ + 10 °C. Values are reported as mean ± SEM (standard error of the mean; n = 3).

At room temperature (~25 °C), the SMP foam samples were compressed until a clear shear fracture appeared near 90% strain. This value was taken as the failure strain, and a 35% compressive strain (~40% failure strain) was used for the following cyclic tests. Permanent plastic deformation was observed at room temperature after loading/unloading compressive tests up to the 35% compressive strain, as shown in Figure 7a. The measured elastic modulus was 2.7 ± 0.12 MPa at room temperature. However, the SMP foam was significantly softened after increasing the specimen’s temperature to its *T*_g_ or higher. The measured elastic modulus of the SMP foam was 0.23 ± 0.017 MPa and 0.18 ± 0.012 MPa at 39 °C and 49 °C, respectively. This mechanical behavior of the SMP foam is critical to its potential role in aneurysm occlusion because the softened SMP foam has a reduced risk of rupturing the diseased blood vessels during the SMP foam’s deployment in an endovascular aneurysm embolization treatment. The elastic modulus values for the foam in this study generally agree with the properties of foams made with CHEM polyurethane (2.69 MPa at room temp., 0.064 MPa above *T*_g_) and polyether polyol-polyurethane (5 MPa at room temp., 0.1 MPa above *T*_g_) [5,35,38]. For both other materials, the SMP foams were tested at *T*_g_ + 30 °C as opposed to *T*_g_ + 10 °C. This could account for the lower elastic modulus values above *T*_g_.

In addition, cyclic compressive tests were used to evaluate the level of strain recovery for shape memory applications. A comparison of the results from the compression testing at *T*_g_ and at 10 °C above *T*_g_ is shown in Figure 7b,c. The SMP foams tested at these two temperature levels showed an initial hysteresis in the first loading/unloading cycle, which could be attributed to the residual stress, material relaxation, or the re-arrangement of dangling chains and side-groups in the polymer. Once the SMP had been fully compressed and relaxed after the first cycle, the hysteresis became relatively insignificant. In addition, the cyclic compressive loading continuously softened the SMP foam, reducing the maximum achievable stress at any given strain. Interestingly, such a reduction in the maximum achievable stress was also observed in the cyclic tensile testing of the dog-bone solid SMP specimens from our previous study [46], whereas a more nonlinear stress reduction was found in our previous cyclic tensile tests. For example, at the 35% applied strain, the measured stress kept reducing after each loading cycle, indicating the potential degradation of the mechanical properties of the synthesized SMP foam. This mechanical behavior will be used to guide the future design and fabrication of the compressed SMP foam as the core component in the intracranial aneurysm treatment.

## 4. Conclusions, Study Limitations, and Future Perspectives

In this study, a novel SMP foam has been developed using aliphatic polyurethane SMPs. We have shown that our SMP foam specimens possess the predicted shape memory function and properties comparable with other similar SMP foam materials. A sugar particle leaching method has been employed to synthesize the porous SMP foam, introducing a new method for manufacturing a foam using an SMP material that is already of interest for aneurysm treatment applications [54]. Qualitative observations have been made to confirm that the synthesized SMP foam is capable of achieving complete shape recovery within a minute of heating past its *T*_g_. The structural properties, such as the average pore size and porosity, have been characterized and found to be uniform in nature. The three-dimensional structure of the material has been recorded using the micro-CT imaging technique, which enables future investigations into computational modeling of the SMP foam’s thermo-mechanical and viscoelastic behaviors. Moreover, an electrical resistance heating method has been employed to activate the shape recovery behavior of the polymer. The relationship between the applied DC current and the surface temperature of the SMP foam has also been examined for more precise control of the shape recovery behavior. Mechanical characterizations of the synthesized SMP foam both at room temperature and above *T*_g_ have been performed, which demonstrates that the material behaves comparably with other polyurethane SMP foams being considered for potential applications in the intracranial aneurysm occlusion. Since fracture was observed at the 90% compressive strain, further investigations would be warranted to increase the compressibility of the foam if the material is going to be optimized for the design and use in the endovascular applications.

There are several assumptions and limitations in this study. First, the monomer content percentages were chosen, according to our previous work [46], to synthesize the SMP specimens with a glass transition temperature of ~39 °C which is ideal for the endovascular embolization treatment. However, there are many different composition possibilities that could result in different *T*_g_ values and different mechanical properties. Future investigations could benefit from characterizations that determine how changes in composition affect the thermo-mechanical properties of the foam.

Secondly, the grain size of cane sugar used in this study, which predominantly governs the pore size of the fabricated SMP foam, varied from 250 µm to 550 µm. Future investigations may be warranted on the sugar grain size and the SMP-sugar ratio to establish a standardized procedure for fabricating the SMP foams with tunable thermo-mechanical performance associated with certain targeted porosity and pore density.

Third, the recommended electric current of 0.15 A, conducted through the carbon fiber wires as a heating element, was determined based on an SMP foam size of 10 mm by 10 mm by 15 mm. A different electric current magnitude may be needed for a different foam size or a different specimen shape. A more in-depth investigation of heat transfer through the SMP foam may be needed to properly control device deployment. In addition, this current was determined based solely on the heating response it produced, which is outside of a physiological setting. Further designs will need to take medical regulations into careful consideration and incorporate safety measures to avoid damaging surrounding tissue via either electric shock or excess heat generation. More refined procedures for the deployment are currently under investigation in our labs.

Finally, moisture has been shown to decrease the *T*_g_ of polyurethane shape memory polymers, which is an effect that could have major implications for applications in the human body [46]. All the mechanical characterization tests in this study were conducted in a standard lab environment open to the air with efforts taken to minimize moisture uptake between synthesis and testing. The water content of the porous SMP after drying was not quantified, but all drying steps were performed identically to normalize any effects of residual moisture. Future tests in an environment more representative of the endovascular conditions are warranted if this material is to be used in the occlusion of intracranial aneurysms. Nevertheless, this study renders a solid foundation to demonstrate the great potential of the developed aliphatic polyurethane SMP foam for the design of novel embolization devices which offer consistent and controllable volume filling ratios during the intracranial aneurysm treatment.

## Appendix A. Micro-CT Imaging of SMP Foam’s 3D Microstructure

In addition to SEM-based quantification of the microstructure of the manufactured SMP foams, a commercial, high-speed, Quantum FX micro-CT system (Perkin Elmer Co., Waltham, MA, USA), equipped with a micro focus X-ray tube with tungsten target and a flat panel detector, was also used to non-destructively image and quantify the 3D microstructural morphology of the synthesized SMP foam samples. The X-ray tube and the detector at the micro-CT system were placed opposite to each other on a gantry that rotates in a counter clockwise around the bed on which the object was placed.

The samples were scanned with an X-ray power at 90 kV and 200 µA, a source object distance (SOD) of 265 mm, a distance to patient of 154 mm, an exposure time of 180 s, and the field of view (FOV) of 30mm x 30mm on the object plane, resulting in a 59 μm resolution with isotropic voxels (512 × 512). Image segmentation and 3D geometry reconstruction of the acquired DICOM image data was then performed in Amira software (FEI Inc., Mansfield, TX, USA) to render the 3D morphological structure. A few representative pores from the 3D images obtained in the CT scan were used to estimate pore-size and roughly cross-check results from the SEM imaging but were not used in any quantitative analysis of the pore size.

## Appendix B. Investigation of Shape Recovery of Pristine Solid SMP Specimen

The shape memory function of the synthesized solid SMPs was also investigated. Each straight beam sample was immersed into hot water (5–10 °C above the expected glass transition temperature), bent up to 180°, and cooled back to room temperature while maintaining the bent shape. Then the SMP sample was placed back to hot water and the material was allowed to return to the initial straight form autonomously. Qualitatively, the pristine solid SMP samples were much more compliant in bending than in either tension or compression. The high degree of flexibility in bending is illustrated in Figure A2. Qualitative analysis of shape memory behavior showed that the samples were difficult to bend without tearing but exhibited a greatly increased compressibility in comparison with the pristine SMP foam.
